# The intersection between logical empiricism and qualitative nursing research: a post-structuralist analysis

**DOI:** 10.1080/17482631.2024.2315636

**Published:** 2024-02-12

**Authors:** Martin Salzmann-Erikson

**Affiliations:** Department of Caring Sciences, Faculty of Health and Occupational Studies, University of Gävle, Gävle, Sweden

**Keywords:** Epistemology, logical empiricism, methodology, nursing research, ontology, postmodernism, qualitative research, research design, social constructivism

## Abstract

**Purpose:**

To shed light on and analyse the intersection between logical empiricism and qualitative nursing research, and to emphasize a post-structuralist critique to traditional methodological constraints.

**Methods:**

In this study, a critical examination is conducted through a post-structuralist lens, evaluating entrenched methodologies within nursing research. This approach facilitates a nuanced exploration of the intersection between logical empiricism and qualitative nursing research, challenging traditional methodological paradigms.

**Results:**

The article focusing on the “what abouts” of sample size, analytic framework, data source, data analysis, and rigour and methodological considerations, challenging the predominance of semi-structured interviews and the reliance on spoken voice as primary data sources, and re-evaluating the conventional notion of “rigour”.

**Conclusions:**

I advocate for a shift from qualitative positivism towards more interpretive and post-qualitative inquiries, this work proposes new trajectories through interpretive, critical, post-qualitative, and artistic turns in nursing research, aiming to transcend positivist limitations and foster a plurality of perspectives and research as praxis. Implications emphasize the need for nursing researchers to expand methodological horizons, incorporating visual and artistic methods to enrich understanding and representation of health experiences, moving beyond positivist norms towards a more inclusive and ethically sound research paradigm.

## Introduction

“How many times do I need to say…?” This phrase may evoke childhood memories. It also serves as a reminder that as parents, we often resort to repetition when teaching our children to adhere to cultural norms and behave appropriately. For me, this exemplifies how logical empiricism intersects language, culture, and our cognitive filters. Originating from the Vienna Circle in the early 20th century, logical empiricism asserts that knowledge is primarily derived from empirical evidence and logical analysis. In this context, the phrase reflects the principle that repetition, akin to empirical observation, can reinforce our beliefs and perceptions, embedding them deeply within our cultural and cognitive landscapes. The more we repeat something, the more it is ingrained in our consciousness, much like the validity granted to empirical data through repeated observation. *If* logical empiricism influences our perception of the world to such a profound extent, *then* it is not unreasonable to suspect its impact on nursing researchers who employ qualitative inquiries. It is worth noting that even the preceding sentence is constructed on the premises of logical empiricism, utilizing conditional statements: if and then. Descartes’ dualism, with its sharp division between mind and body, has profoundly influenced the Western ontological perspective, particularly in healthcare. This rigid bifurcation has manifested in various dichotomies such as body/mind and patient/staff, shaping healthcare’s approach to treatment and understanding of health. However, this dualistic view has faced substantial criticism for its oversimplified and reductionist nature, often failing to acknowledge the complex interplay between mental and physical health. As we delve into the intersection of logical empiricism and nursing research—nurses learn research methods to do nursing research themselves–, it becomes imperative to challenge and re-evaluate these Cartesian divisions. Such ontology oversimplifies the human experience and impede a more holistic, integrated approach in healthcare, which recognizes the intricate connections between mental and physical well-being.

### Positivism and post-positivism in nursing research

Positivism asserts that authentic knowledge emerges through empirical and logical methods, underscoring the importance of observable data and empirical evidence. It values objectivity, the quantification of phenomena, and the pursuit of generalizable findings via standardized methodologies, positing that reality is objective and can be understood scientifically (Keuth, 2015). As explained in a review of paradigms in nursing (Weaver & Olson, 2006), positivist research aims for control and prediction, and theoretical knowledge is viewed as an absolute entity. Expanding on the tenets of positivism, logical empiricism presents a refined perspective that underscores the synergy between empirical observation and logical analysis in the construction of knowledge. This principle signifies a departure from traditional positivism by asserting that empirical data must be complemented with logical reasoning to form a coherent scientific understanding (Radder, 2021). Furthermore, logical empiricism recognizes the limitations of sensory experience and emphasizes the role of theoretical language in scientific discourse, thus bridging the gap between empirical observation and theoretical constructs (Keuth, 2015).

In contrast, post-positivism embraces a more contextual approach and questions the neutrality of positivism, acknowledging human limitations in observing and understanding phenomena. It perceives truth as conditional, influenced by various factors, and asserts that knowledge is provisional, open to further investigation. Post-positivism seeks truth as a probable value, contrasting with the positivist view of an absolute truth (Tanlaka et al., 2019; Weaver & Olson, 2006). Post-positivism does not reject “scientific methods” (randomization, sample size calculation, and statistical analysis) but approaches them with an understanding that truth is not solely an external, objective entity. Rather, post-positivism situates truth within specific contexts, recognizing that our understanding of reality is influenced by the circumstances in which knowledge is constructed and interpreted. This marks a departure from positivism’s absolute certainties, favouring a more probabilistic understanding of knowledge (Weaver & Olson, 2006).

Post-positivism is frequently employed in nursing research, as illustrated by a study by Ye et al. (2024) aimed at assessing the efficacy of stepwise swallowing training in patients with Alzheimer’s disease. This study heavily relies on empirical observation and measurement, a hallmark of both positivism and post-positivism. It employs standardized tools to evaluate the efficacy of stepwise swallowing training. Utilizing a randomized controlled trial (RCT) format, which involves hypothesis testing with control and intervention groups, is indicative of a post-positivist approach. While positivism often involves straightforward observation and measurement, post-positivism typically accepts that knowledge is conjectural and employs hypothesis testing to approximate the truth. The analysis acknowledges the probabilistic nature of the findings, as indicated using statistical tests of significance and confidence intervals. This suggests an acceptance that conclusions are not absolute but are the best approximation of reality based on current evidence. This aligns with post-positivism’s acknowledgement that all observations and conclusions are fallible and subject to revision. Post-positivism critiques positivism for its rigid adherence to objective truths, advocating for a more nuanced understanding that embraces the complexity and subjectivity inherent in health research (Clark, 1998; Corry et al., 2019; Weaver & Olson, 2006).

### Interpretivism in nursing

Advancing through the evolution of paradigms, interpretivism emerged as a counter-response to post-positivism, challenging its emphasis on objectivity and reliance on empirical evidence. Interpretivism expands understanding by delving into the subjective and constructed nature of reality, emphasizing the significance of personal experiences and social contexts in shaping knowledge (Ryan, 2018). It centres on understanding human experience and social context and emphasizes the subjective nature of reality, viewing knowledge as constructed through interactions and interpretations. In the field of nursing research, the interpretivist paradigm has gained increasing traction. It recognizes the value of various methodologies, among which phenomenological hermeneutical studies are one prominent approach but not the sole method. This is exemplified in a study by Davidson et al. (2024), which utilizes a phenomenological hermeneutical approach to deeply investigate the personal and subjective experiences of parent-caregivers of children with rare diseases. The study delves into the intricate emotional and practical realities of caregiving during the challenging postpartum period. Utilizing interpretative phenomenological analysis (IPA), the research revealed nuanced themes such as the shift in parental identity, the transformation of family dynamics, and the process of adaptation and adjustment to a “new normal.” These findings underscore the interpretivist emphasis on deep, contextualized understanding of individual experiences, particularly in situations marked by significant life changes and medical complexities.

Another example is a Finnish study on nursing students’ ways of experiencing writing a bachelor’s thesis exemplifies interpretivism through a phenomenographic study. It focuses on the diverse subjective experiences of nursing students at a pivotal stage of their academic journey. This study delves into how students navigate the complexities of thesis writing, revealing a range of experiences from solitary structuring to collaborative understanding and transformation. By capturing these different perspectives, the study highlights the interpretivist emphasis on individual perceptions and their impact on learning processes (Henttonen et al., 2023).

### Critical theory in nursing

Moving beyond the confines of interpretivism, critical theory find limitations in interpretivism for its focus on individual experiences without sufficiently addressing broader social, political, and power structures. Critical theorists argue that interpretivism, while valuable in understanding personal perspectives, often overlooks how these experiences are shaped by and contribute to systemic inequalities and societal constructs. This perspective advocates for a more comprehensive analysis that includes the examination of societal power dynamics and their impact on individual experiences (Ekström & Sigurdsson, 2002).

Furthermore, McGibbon and Lukeman (2019) advocate the importance of adopting critical perspectives in nursing, emphasizing the significance of these perspectives in the realm of social justice and the urgent need for transformative action. Illustrating this, the research by Paradis‐Gagné and Pariseau‐Legault (2021) utilized critical ethnography to highlight the crucial role of nursing practices in challenging social inequalities and fostering empowerment among marginalized communities, as demonstrated in their study focusing on individuals experiencing homelessness. This methodology championed transformative knowledge, emphasizing the necessity for societal and healthcare reforms to address systemic injustices and promote social justice. Paradis‐Gagné and Pariseau‐Legault encapsulated a call for nursing to not only understand but actively challenge the underlying structures of oppression and inequality in healthcare settings.

### Post-structuralism in nursing

Shifting the focus, post-structuralism presents a critique of critical theory, particularly its dependency on broad structures and overarching narratives to understand societal dynamics. From an ontological and epistemological standpoint, post-structuralism argues that critical theory, despite its focus on power and social constructs, still operates within fixed structures and binary oppositions. Post-structuralism challenges this by deconstructing these structures and narratives, emphasizing the fluidity and multiplicity of meanings, identities, and experiences (Harcourt, 2007). In nursing, Holmes and Gastaldo (2002) delved into the Foucauldian concept of governmentality to analyse nurses’ role in societal governance. They argue that nursing practices are not merely acts of care but are intricately linked to political and power structures. This perspective is crucial in understanding how nursing, often perceived as a discipline focused on individual care, is implicated in broader social and political dynamics. Holmes and Gastaldo emphasized the need for nursing to recognize and critically engage with its role in these dynamics, particularly in relation to the governance of populations and the production of societal norms and behaviours. This approach aligns with the critical perspective’s call for a broader analysis of power relations and their impact on health and illness.

In the article by Melamed (2023), post-structuralist feminist analysis was applied to examine the use of electronic foetal monitoring (EFM) in labour, specifically the cardiotocograph. This approach critiques the traditional medical model of childbirth, highlighting how EFM can symbolically disrupt the intrinsic relationality between mother, foetus, and midwife. It emphasizes the philosophy of self as relational and semi-permeable, challenging notions of sovereign individuality and drawing attention to the collaborative nature of birth. This perspective is significant for nursing as it reorients the understanding of childbirth from a mechanistic, individualized process to one deeply embedded in interpersonal and social dynamics, encouraging a shift towards more holistic, relationally-focused care practices.

Post-structuralism is not merely a divergence from traditional methodologies but a response to the intricate subtleties of interpretive research. It represents an emancipation from the hegemonic biomedical discourse, heralding a new era in nursing research that embraces diverse, inclusive, and innovative methodologies. This evolution marks a significant stride towards a more holistic understanding of health care, transcending the limitations of established paradigms. Therefore, the purpose of this article is to shed light on and analyse the intersection between logical empiricism and qualitative nursing research.

#### Reevaluating epistemologies in nursing research

Denzin et al. (2006) argue that qualitative research transcends mere methodology, being deeply interwoven with political concerns. These concerns significantly influence which research questions are posed, the methods of investigation, and the interpretation and dissemination of findings. Consequently, nursing researchers possess the capability to amplify often marginalized voices, thus demonstrating a commitment to inclusivity within healthcare narratives. This is evident in research like Mattsson et al. (2023) study on women experiencing homelessness during the pandemic, or Malebye et al. (2023). exploration of family experiences in psychiatric hospitals in South Africa. Such research aligns with interpretive epistemologies that prioritize “lived experience” and “authentic voice.” However, post-structuralists like Grant (2014) challenge this, arguing against fixed, objective truths, and advocating for an analysis of how knowledge is constructed by power and social structures. St. Pierre (2023), drawing on Foucault (1971/1972), moves beyond this dichotomy, advocating for a post-structural approach that critically interrogates traditional research methodologies. This approach questions the assumed stability of empirical narratives and encourages a reimagining of qualitative inquiry, resonating with nursing theories by Parse (1999) and Newman (1993), which emphasize the fluid nature of human existence. While nursing has not predominantly evolved from a post-structuralist perspective, its methodologies have been significantly influenced by positivist ontology and epistemology, highlighting the need for a more nuanced and dynamic approach in nursing research.

### What about our dear methods?

As we navigate the complexities and uncertainties inherent in our understanding of knowledge and being, it becomes crucial to critically evaluate and question the research methodologies that we habitually rely upon. In this regard, we can draw insights from Foucault (1997) and his exploration of “methods”:

I do not have a methodology that I apply in the same way to different domains. On the contrary, I would say that I try to isolate a single field of objects, a domain of objects, by using the instruments I can find or that I forge as I am actually doing my research, but without privileging the problem of methodology in any way. (pp. 287–288)

Foucault, in his critical examination of social sciences, argued against the rigid and often reductive nature of standardized research “methods.” He contended that such methods can inadvertently constrain our understanding of complex social phenomena (Foucault, 2002). Foucault’s critique was rooted in his broader philosophical position that knowledge is deeply intertwined with power structures, and that standardized methods can perpetuate certain power dynamics, potentially overlooking diverse or marginalized perspectives (Foucault, 1995). His argument was not against the use of methods per se, but against their uncritical application without considering their implications in the context of power-knowledge relationships (Foucault, 2002). Echoing this critique, St. Pierre (2023) advocates for a post-structural approach to qualitative research, challenging the conventional methodological adherence and promoting a more reflexive and dynamic practice. This approach aligns with Foucault’s critique, emphasizing the fluid and contingent nature of knowledge and understanding in social research. However, there often seems to be a disproportionate focus on the methodological aspects, sometimes overshadowing the substantive outcomes and interpretations of the research. This phenomenon, as Patai (1994) points out, can lead to method becoming a display of power, while Bauwens et al. (2013) emphasizes the overlooked significance of paradigms and their impact beyond mere methodological sections. Similarly, Nader (2018) cautions that an overemphasis on methods can eclipse the fundamental purpose of critical thinking in humanities and social sciences. Drawing upon Foucault’s ideas (1995), these academic debates can be viewed as mechanisms of panoptic disciplinary power, where the methodological rigour is often scrutinized more than the insights it brings.

### Post-structuralist perspectives on knowledge production and objectivity

The predominance of evidence-based knowledge, deeply rooted in positivist philosophy, often gives precedence to empirical and measurable data. This approach can inadvertently sideline or undermine the legitimacy of various forms of knowledge production, particularly those integral to qualitative research. Such a hegemonic stance is critiqued as a form of “disciplining” qualitative studies, leading to the marginalization of qualitative methods and restricting the exploration of complex social phenomena that are not easily quantifiable or empirical (Denzin et al., 2006). While a post-structural analysis indeed emphasizes that the past is not merely a backdrop but an active element shaping the present, it is important to acknowledge that this perspective is not exclusive to post-structuralism. For example, interpretivism and critical theory also recognize the dynamic interplay between history and the present. What distinguishes post-structuralism, however, is its particular emphasis on how power structures and discourses within historical contexts influence current understandings and social realities (Patton, 2015; Ryan, 2018). Consequently, it becomes imperative to integrate history in order to comprehend the influence of logical empiricism in qualitative nursing research. It can be briefly noted that in the early stages, emphasis was placed on describing and interpreting the experiences of patients, relatives, the nursing profession, and nursing students through case studies and participant observation (Peplau, 1952; Thompson & Sutton, 1985). Grounded theory is a research methodology that involves developing theory through the collection and analysis of data, where the theory emerges from the data itself, rather than being imposed on it beforehand. This approach emphasizes inductive reasoning and the iterative process of data collection and analysis (Charmaz, 2014). Phenomenology, on the other hand, is a qualitative research approach that seeks to understand and describe how people experience a certain phenomenon, focusing on the subjective meaning and interpretations of human experiences (Van Manen, 2016). Nursing researchers appealed to robust methods such as Grounded Theory and phenomenology (Haase, 1987; Hinds, 1984), even though they were originally developed in other subject areas, like social sciences and anthropology. However, despite their epistemological compatibility with nursing, Thorne (1991) noted some challenges for nurses who adopted methods developed in other disciplines.

nursing simultaneously values the uniqueness of each individual and the commonalities that may be contained within each human experience. […] Thus, research methods to uncover the nature of tacit everyday reality or ontology will not provide us with a clear direction beyond their classification of the basic beliefs that drive out practice. Their methods may seem familiar to us, but that familiarity may be produced of our inherent respect for subjective experience rather than a reflection of the answers such methods will produce for knowledge development. (p. 189)

In Thorne’s (1991) discussion article on methodological orthodoxy, she posits that nursing researchers ought not to engage in deeply philosophical-rooted methodologies or indiscriminately mix methods. Instead, she advocated for a flexible approach, referred to as methodological heterodoxy—a departure from the conventional, dominant or accepted methodologies—, which separates the orthodoxy found in well-established methods from the social and anthropological sciences, such as Grounded Theory, phenomenology, and ethnography (Thorne, 1991; Thorne et al., 1997). She argued that, for example, nurses’ use of anthropological methodologies might share ontological values, but nursing research questions are often limited to a specific part of a cultural setting. Her critique was that, contrary to anthropological assumptions, nurses do not study culture as a whole, leading to inadequate interpretations that fail to consider the broader cultural context (Thorne, 1991). Several nursing research studies tend to narrow their focus to research questions that pertain to specific aspects of culture or particular social activities. Similarly, ethnographic work of nurses has been conducted diligently during the last decades; for example, Belle et al. (2020) explored person-centred care in hospital wards using ethnographic methods. Even more detailed and “tweezer-likened” versions of ethnography is seen in nursing research: *focused ethnography*. For example, focused ethnography has been used to investigate factors influencing the practice of newly graduated nurses in acute care settings (Charette et al., 2019). The purpose of citing these studies is not to diminish their methodological axiology, but rather to demonstrate the significance of Thorne’s (1991) ideas; she wrote: “While the culture concept represents part of what we want to understand, it is never the whole. Our enterprise is therefore somewhat more ‘fuzzy’ than of true ethnography.” (p. 190).

Thorne et al. (1997) posited that while quantitative methods may not adequately address subjective experiences, qualitative methods developed in other subject areas may not be appropriate for nursing research questions or the researcher. Thorne (2016) distinguished between nursing research, which is intended to inform practice and develop applied knowledge relevant for nursing values, and social science research, which is focused on developing and refining theory. Despite the shared ontological aspects between social science and nursing science, there exists a divergence in disciplinary orientation and epistemology—or, at least, this is commonly believed. Thorne aimed to develop a nursing-oriented method that was not bound by epistemological orthodoxy, like separating the church and state; although Thorne did not use that analogy.

Sandelowski (2000) published an article titled “Whatever happened to qualitative description?” In this article, she proposed qualitative description as an alternative “method that researchers can claim unashamedly without resorting to methodological acrobatics.” (p. 335). One possible explanation for this preference is that assigning a label or a name to a method itself imposes certain expectations and obligations on the inquiry. It is crucial to acknowledge that neither Sandelowski (2000) nor Thorne (1991) present methods as cookbook-like recipes; rather, they provide frameworks for conceptualizing and approaching methods.

The core concern may lie in the hegemonic position held by researchers who assert that something exists and can be accurately described. According to this perspective, the story of a study participant is never fixed or final; it is an ongoing process of becoming, with multiple interpretations continuously emerging: “one interpretation piled on another and another” (St. Pierre, 2023, p. 27). Consequently, the search for knowledge can never truly reach a definitive destination. Adopting a post-structuralist viewpoint, it becomes essential to consider the contextual situation in which something is observed.

### Caught in the post-positivist paradigm

Ingress here about how nurses have one foot in post-positivism and the other in interpretivism. The fallacy of doing qualitative and interpretive research but really not. To illustrate this, we will use domains of qualitative research, sample size, etc.

### What about sample size?

How many participants or interviews should be included in qualitative inquiries? When is it considered sufficient? Should the decision be made prior to data collection or during the research process? Saturation is a concept frequently employed in qualitative research to signify the point at which no new data emerges from interviews (Bowen, 2008). Grounded Theory studies often involve a larger number of participants and interview hours compared to other methods (Marshall et al., 2013). This idea challenges the epistemology rooted in logical empiricism, which often prioritizes large sample sizes for external validity. Thorne (Thorne, 2020) in a later editorial, questioned the applicability of data saturation, suggesting it can be misleading in the context of qualitative research. This aligns with the perspective of Chicoine et al. (2022), who argued that in qualitative studies, the pursuit of data saturation is less relevant compared to obtaining a deeper understanding of participants’ experiences. They emphasized the importance of rich data that adequately answers the research question, rather than adhering strictly to sample size considerations. This discussion reflects a critical evaluation within the qualitative research community, focusing on the depth and quality of data rather than the quantity, a fundamental departure from the empirical emphasis of logical empiricism.

The issue of a small sample size is particularly relevant in psychiatric and mental health nursing research, one of my own research areas, as recruiting participants for qualitative interviews can present severe challenges. This aligns with my own experience of recruiting participants for inclusion in a study on former patients’ experiences of being admitted to a psychiatric intensive care unit (Salzmann-Erikson & Söderqvist, 2017). In that study our original plan was to collect data from about twenty individuals but only succeed to include four individuals even though we tried for two and a half years. Presumptive participants may be hesitant to participate due to the stigma associated with mental illness, fear of disclosing personal information, or lack of trust in the research process. Additionally, many individuals with mental health conditions may experience symptoms such as apathy or disinterest, making it challenging to engage them in research. Furthermore, some individuals may not have access to appropriate resources making it difficult to connect with them, for instance, women who lives in homelessness (Kneck et al., 2022).

### Analytic framework

Thorne (2016) informs that it is not appropriate, in nursing research, to enter a study without any preunderstandings—in “*tabula rasa* (blank slate)” (Thorne, 2011, p. 446)—or without theoretical inquiries, because of the close bonds nursing researchers have to their own clinical experience and professional knowledge and to the specific study aim. Similarly, Sandelowski (2000) argued that qualitative description is the least theoretical approach. However, in a later publication (Sandelowski, 2010), she clarified that her original perspective had been misinterpreted by authors who cited her work, suggesting that qualitative description does not involve theory at all. Sandelowski cited Haraway (1991) to emphasize her disagreement with such interpretation, stating, “There is no such thing as a view from nowehere” (Sandelowski, 2010, p. 80). In a theoretical discussion paper, co-authored by Thorne (Chiu et al., 2022), the authors reasoned about the value a theoretical framework may have on the outcomes of an inquiry. A wide range of theoretical framework are used by authors who have used Interpretive Description in nursing studies. Adopting an eclectic analytical framework in nursing research can lead to a more comprehensive understanding of complex inquiries. In this context, eclecticism involves integrating a wide array of theories and methodologies to enrich analysis and interpretation. Such an approach offers a holistic perspective on the research topic. For instance, in a Grounded Theory study by Göras et al. (2023), the escalation process of intensive care during the pandemic was examined. The researchers applied concepts from complexity science, viewing the settings as chaotic, resilient, and adaptive systems. While this theoretical lens has gained recognition in nursing research, it is important to note that chaos and complexity theories originally derive from meteorological science and mathematics (Braithwaite et al., 2017; Kauffman, 1995). This interdisciplinary borrowing illustrates the value of eclecticism in providing nuanced insights into nursing phenomena. Meanwhile, eclectic analytical framework may not be the golden standard. While it provides a breadth of perspectives, ensuring coherence and depth in the research methodology a critical consideration remains; especially if we want to claim a more genuine anti-oppressive research stance. From a post-structuralist perspective, we also need to acknowledge the intersectionality of culture, history, and non-static identities, as well as the idea of social constructions (Grant, 2014; St. Pierre, 2023). Through the lens of a framework, we may comprehend, gain insights, or become informed by a study’s results slightly differently. Thus, a framework can add additional value to a study that extends beyond mere reports of participants’ spoken words, often dressed in captivating phrases such as *codes* or *meaning units*. In this spirit, we need to critically examine the values that frameworks can add to our qualitative and interpretive inquiries and post-structuralist works, as this relates to research axiology.

### What about data source?

In a brief search conducted in the Cumulative Index to Nursing and Allied Health Literature (CINAHL), I found that there were nearly 95,000 records for the search term “semi-structured interviews” and a similar number of records for “focus group” when search individually. On the other hand, the search for “participant observations” resulted in approximately 19,000 records. It is apparent that *talking to* individuals is the most commonly used data source in qualitative nursing research. There is a prevailing norm that places significant value on the spoken words of study participants. However, St. Pierre and Jackson (2014) raised critical question about the glorification and the added value derived from interviews:

words spoken by participants are privileged regardless of their adequacy to respond to the study’s substantive and theoretical demands […]. If a researcher is studying prisons, for example, why would she not include as data Foucault’s (1975/1979) words in his book, Discipline and Punish: The Birth of the Prison, which is one of the most famous studies of prisons? Why are Foucault’s words consigned to the literature review? Is it because they are written, textualized? However, that criteria cannot hold because words spoken in face-to-face interviews do not count as data until they are written, textualized in interview transcripts. (p. 716)

Nursing researchers often favour semi-structured interviews, a preference influenced by an epistemological orthodoxy that leans towards the belief in an objective way of knowing and understanding the world. This approach is predicated on the assumption that participants’ spoken words provide a direct, unmediated access to truth and meaning. However, as Haraway (1988) critiques, this belief can be seen as an illusion implying a false sense of objectivity in understanding human experiences. It overlooks the complexity and subjectivity inherent in human narratives and assumes a direct correlation between language and lived reality. The tendency to privilege voice and rely on spoken words as a direct access to truth often leads to an underestimation of the complexities and contextual factors shaping individual stories, as highlighted by Jackson and Mazzei (2009). Echoing this sentiment, Grant (2014) critically examines the concept of “authentic voice”, noting that “The light of human meaning is always refracted through the dark glass of language, and language is always unstable.” (p. 545). This perspective underscores the need for a more critical approach to the rationale behind conducting interviews, encouraging researchers to question and articulate their methodological choices more explicitly. St. Pierre’s lamentation, “We must have hundred of articles on interviewing—it’s insane. I think we’ve created a monster” (Guttorm et al., 2015, p. 16), starkly illustrates the proliferation and potential oversimplification of qualitative methodologies, suggesting a deviation from their original, critical intent. It behoves nursing researchers to transcend the procedural orthodoxy that has characterized qualitative inquiry, fostering an environment where methodological innovations are not only encouraged but are seen as essential to capturing the multifaceted nature of human experience. This shift necessitates a deliberate move away from formulaic approaches, advocating for a methodological pluralism that embraces the complexity and unpredictability inherent in human narratives.

However, shifting towards a performative orientation, as discussed by Iregbu et al. (2022), allows for a more dynamic understanding. This perspective recognizes knowledge as an evolving process of meaning-making, challenging the notion that truth is a static entity simply awaiting discovery in interviews. Thus, it becomes clear that the spoken words of participants should not be seen as holding a privileged position in accessing truth and meaning, but rather as one of many elements in a continuous process of knowledge construction. Over thirty years ago, Thorne (1991) called for methodological heterodoxy. So, what are the ways to recover from this “epistemological navel-gazing?” One potential avenue for future research is to delve further into discourses within digital environments, as these spaces provide a rich and dynamic context for understanding contemporary social interactions and narratives. Additionally, employing embodied mapping as a methodology offers a unique approach. According to Rieger et al. (2022), this method “moves beyond methods and even theory-as-method as it becomes an entanglement of doing and becoming undone.” (p. 2). A third example is the emergence of performative approach engaged artistically and creatively with the data, allowing it to guide our interpretive process (Iregbu et al., 2022). This may involve composing music, dancing, drawing, or other artistic methods that capture dynamic aspects of the phenomenon beyond language. Centering the body and affect in data collection and analysis fosters the emergence of new perspectives, rather than capturing pre-existing meanings.

### What about data analysis?

Elliott (2018) described coding as “an almost universal process in qualitative research; it is a fundamental aspect of the analytical process“(p. 2850). More detailed coding practices, such as line-by-line coding, are taught in methods books and articles. In the article entitled “The art of coding and thematic exploration in qualitative research” Williams and Moser (2019) explained that “each textual line of an interview or document is scrutinized with the goal of maintaining the researcher’s focus on the text” (p. 51). Strategies have been developed, such as intercoder reliability, to ensure agreement among multiple researchers analysing the same qualitative data (MacPhail et al., 2016). St. Pierre (2023) challenges the significance of coding, dismissing it as a product of the methodological business machine and referring to it as a “quasi-statistical analytical practice” (St Pierre & Jackson, 2014, p. 715). St Pierre (2023) explains that interpretations of data are continuously layered upon previous interpretations, rendering the act of coding as a perpetuation of this process. So, why do we continue to code data as if we were qualitative positivists (Eisenhardt, 1989) or transcendental realist (Miles & Huberman, 1994)? It would be ironic within the context of this article to provide a definitive cause. As a nurse, I believe that our historical struggle for power within healthcare systems, particularly against the dominance of physicians and the medical hegemony, has shaped our inclination to adopt coding as a means of achieving legitimacy in research. Perhaps this analysis aligns with the concept of nursing diaspora. However, it is important to note that this is a subjective and transient analysis from my current standpoint.

### What about rigor and methodological considerations?

While certain data collection techniques, such as interviewing and observing, may exhibit similarities between quantitative and qualitative research, the question of what confers legitimacy and quality to research has long been a contentious topic. Lincoln and Guba (1985) proposed an alternative to the positivist notion of validity and reliability in qualitative research by introducing the concepts of credibility, dependability, confirmability, and transferability as indicators of trustworthiness. Various techniques have been described in the literature to ensure rigour in qualitative research, such as respondent validation or member checking, peer debriefing, and audit trials, among others. However, it could be argued that these techniques can be seen as deceptive or illusory in their ability to guarantee quality in qualitative research. If we approach rigour from an interpretive or post-structuralist standpoint, it becomes apparent that the concept of rigour often assumes adherence to methodological orthodoxy; keep in mind Foucault’s (1997) approach to the use of methods, which challenges the notion of rigid methodological frameworks: “I do not have a methodology […] I am actually doing my research, but without the privileging the problem of methodology” (p. 287–288). Therefore, when employing a specific method, we are compelled to acknowledge the inherent duality of strengths and weaknesses in our studies, often discussed in sections such as Methodological Considerations or Limitations. Upadhyay (2014) put it “attempts to apply rigor, validity or reliability to qualitative research have the result of accepting a positivist framework based on a quantitative assumption that belittles the worth of qualitative research per se.” (p. 68).

#### The problem with member checking

Member checking is a process where researchers return to participants after data analysis to share findings, verify their accuracy, or gather additional insights. It has been recognized in the research community as a gold standard for establishing credibility and ensuring rigour (Lincoln & Guba, 1985; Motulsky, 2021). This practice is commonly employed in nursing research as a means to affirm credibility and maintain rigour (Chicoine et al., 2022; Olawo et al., 2021; Slomp et al., 2022). However, Rolfe (2006), contends that member checking may be at odds with qualitative epistemology. He argues that expecting agreement among respondents or experts on the same categories and themes is unrealistic. From a positivist perspective, verifying the correctness of interpretations makes sense. Yet, through a post-structuralist lens, member checking implies a hierarchy that necessitates the validation of the researcher’s interpretations by participants, or vice versa. Such a practice can reinforce power dynamics and overlook the collaborative nature of knowledge construction. Additionally, since participant perspectives can evolve, the concept of a stable, confirmable “truth” becomes problematic. Post-structuralism acknowledges that understandings are dynamic and context-dependent. Furthermore, it views language, as used in interviews and member checking, as an unstable medium for conveying experience and reality, casting doubt on its ability to accurately capture and validate experiences (Foucault, 2002; St. Pierre, 1999).

### What about quality in interpretive and post-structuralist inquiries?

Despite post-structuralism’s rejection of many commonly accepted aspects of the research process, it is important to note that post-structuralist inquiries still strive for good quality. However, the concept of what constitutes good quality varies, challenging us to adopt a different perspective. Some endeavours have been documented in the literature to address this matter. For instance, Madill et al. (2000) came close to post-structuralism by outlining quality criteria in the context of radical constructivism, an epistemology that shares many tenets with post-structuralism. They suggest that internal coherence can be utilized as a criterion, defined “an evaluation of the extent to which the analysis ‘hangs together’ or is non-self-contradictory” (Madill et al., 2000, p. 13). This implies the analysis’s capacity to challenge and transform traditional practices, discourses, and power dynamics that define the nursing metaparadigm. Emancipatory potential in nursing research seeks to liberate the four central concepts—person, environment, health, and nursing—from rigid or reductionist interpretations, encouraging more holistic, person-centred, and culturally sensitive approaches to understanding and practicing nursing. What about polyvocality? It pertains to the ability to reflect and respect the diverse voices and perspectives within the nursing metaparadigm. This includes not only the voices of nurses and patients but also those of family members, communities, and other healthcare professionals.

### Implications for nursing researchers

Given that a substantial portion of qualitative nursing research relies on semi-structured interviews, there is a prevalent belief that participants’ spoken language, as fixed subjects, can convey their experiences and epiphanies as objective truths inscribed in history. To move beyond this approach, more nursing researchers should develop their post-structuralist literacy (Petrovskaya, 2022). This would enable to expand beyond traditional research confines and better align with society’s evolving context, where their research is embedded. Petrovskaya’s encouragement for nurse researchers to deepen their post-structuralist understanding is a call to critically reflect on how logical empiricism influences thinking, research formulations, and analyses in interpretive inquiries. The epithet “post-structuralism” is far from neutral; thus, nursing researchers might find it more appealing to use the term “post-qualitative inquiries” (St. Pierre, 2021, 2023). To St. Pierre, post-qualitative inquiries signifies a critical departure from traditional qualitative research methodologies, advocating for a methodological revolution that embraces complexity, uncertainty, and the non-linearity of human experiences (Guttorm et al., 2015). In an article co-authored by St. Pierre, the authors critically engage with the hegemonic Eurocentric biases pervasive in academic research (Stewart et al., 2021). They advocate for the adoption of methodologies that are not only inclusive and ethically sound but also instrumental in deconstructing entrenched power dynamics. This approach facilitates a representation of participant narratives in their full complexity, consciously avoiding the pitfalls of oversimplification and stereotypical portrayals. Their work epitomizes the essence of post-structuralist thought, challenging normative assumptions and advocating for a nuanced understanding of diverse experiences within research contexts. Drawing on Thorne’s (1991) earlier insights, “instead of platitudes about individualizing care or being more culturally sensitive, analysis could be directed toward the social and cultural contexts in which individual experiences is constructed.” (p. 194). Nursing researchers have started embracing visual methods, such as photo-voice (Boamah et al., 2023) and photo-elicitation techniques (Lood et al., 2023), to glean deeper insights from patients and communities. These methods, which involve participants creating photographs related to their health experiences for discussion during interviews, uncover subjective meanings that words alone may not fully express. While these photo-based techniques are promising, exploring even more diverse artistic methods could further push the boundaries of nursing knowledge. Alternative artistic methods offer new trajectories for nursing research (cf. Thorne, 1991). Thorne’s early call for attention to broader social and cultural forces in health experiences remains relevant. Arts-based approaches, such as poetic inquiry (Chambers, 2023; Green et al., 2021), ethnodrama (Lewis et al., 2023), visual art (Skukauskaite et al., 2022), and digital storytelling (Munajah et al., 2022) offer innovative ways to generate and represent insights. By embracing these creative methodologies, nursing research can challenge the conventional word-centric academic narrative (cf. Jackson & Mazzei, 2009) and place the person at the centre of research through diverse meaning-making mediums. Emphasizing aesthetic, sensory, and embodied knowledge alongside cognitive data enriches nursing research; moving past the enduring positivist norms in nursing research is crucial for fulfilling our societal ethical duty. Nurse researchers need to broaden the focus from solely clinical settings to consider the wider socio-political factors that shape health (cf. Mattsson et al., 2023). Recognizing how systemic forces like economics, politics, culture, and geography influence community health and write futures of health or illness is key to a comprehensive understanding of well-being in nursing practice (Adeline, 2000).

Academic writing that may limit conceptualizations as it keep the person at centre by honouring diverse mediums for meaning-making. Nursing research will benefit by valuing aesthetic, sensory, and embodied knowledge on par with cognitive data. Moving beyond the lingering positivist norms in nursing research is essential for us to fulfil our ethical duty and responsibility to society. For too long, nursing researchers have narrowly constrained our focus to the clinical environment and individualistic conceptions of health. Yet the conditions shaping human wellbeing extend far beyond the hospital walls. Health is intimately tied to the broader socio-political landscape that impacts people’s daily lives. As nurses, we must break out of the “clinical gaze” and attend to how systemic forces—economics, politics, culture, geography—imprint upon communities and write futures of health or illness.

Emancipation from positivism’s limiting legacy is required to equip nursing researchers with the tools to analyse and act upon the upstream structural determinants of health. The research questions must interrogate the distribution of power and resources that pattern vulnerability and access in society. And the methodologies should illuminate the historical, ideological and material contexts that give rise to health inequities. While the post-qualitative turn does not inherently prescribe an ethical orientation, it provides a flexible framework that can be applied to nursing research aimed at social justice, encouraging us to reconsider how knowledge is produced and whose voices are prioritized. But we have yet to actualize the full potential of “research as praxis”. Too often, those most burdened by injustice are excluded from shaping the research agenda or participating on equal terms. As nursing researchers, we must centre community priorities and cede some ground to marginalized voices. This demands relinquishing the privileged position as “expert” and embracing solidarity-based co-inquiry. Such an orientation recognizes that people with lived experience hold deep insights about improving health within their own reality. Building partnerships, not extracting data, should be the guiding ethos for nursing research committed to social change. Our goal must not be knowledge for its own sake, but galvanizing collective action.

## Authorships

Martin Salzmann-Erikson is the only author of this manuscript.

## Writing assistance

Use of AI assistance: *During the preparation of this work, the author employed the service of ChatGPT September 25 Version, to scrutinize grammar and spelling, and to rephrase sentences for enhanced clarity, like the functions previously performed by a human language editor, given the author non-native English language background. Following the utilization of this service, the author meticulously reviewed and amended the content as necessary and assume full responsibility for the content of the publication. No analyses were formulated with the aid of artificial intelligence*.

## Use of AI assistance

During the preparation of this work, the author employed the service of ChatGPT September 25 Version, to scrutinize grammar and spelling, and to rephrase sentences for enhanced clarity, like the functions previously performed by a human language editor, given the author non-native English language background. Following the utilization of this service, the author meticulously reviewed and amended the content as necessary and assume full responsibility for the content of the publication. No analyses were formulated with the aid of artificial intelligence.
